# Choice Quality as a Function of Decision Accuracy and Search Cost

**Published:** 2019-07

**Authors:** Reza Rastgoo Sisakht, Shabnam Mousavi, Rahimeh Negarandeh, Hamid Valizadegan, Maryam Noroozian, Mehdi Tehrani-Doost, Emran Mohammad Razaghi

**Affiliations:** 1 Department of Neuroscience and Addiction Studies, School of Advanced Technologies in Medicine, Tehran University of Medical Sciences, Tehran, Iran.; 2 The Johns Hopkins Carey Business School, Johns Hopkins University, Washington DC, United States of America.; 3 Médecins Sans Frontières, Tehran, Iran.; 4 Decision Science and Knowledge Engineering, Behsazan Mellat Co, Tehran, Iran.; 5 Division of Memory and Behavioral Neurology, Department of Psychiatry, Roozbeh Hospital, Tehran University of Medical Sciences, Tehran, Iran.; 6 Department of Neuroscience and Addiction Studies, School of Advanced Technologies in Medicine, Tehran University of Medical Sciences, Tehran, Iran; Department of Psychiatry, School of Medicine, Research Center for Cognitive and Behavioral Studies, Tehran University of Medical Sciences, Tehran, Iran.; 7 Department of Psychiatry, Roozbeh Hospital, Tehran University of Medical Sciences, Tehran, Iran; Department of Neuroscience and Addiction Studies, School of Advanced Technologies in Medicine, Tehran University of Medical Sciences, Tehran, Iran.

**Keywords:** *Cost-Benefit Calculations*, *Decision Process*, *Expected Value*, Mouselab* Quality of Choice, *Utility*

## Abstract

**Objective:** A prominent challenge in modeling choice is specification of the underlying cognitive processes. Many cognitive-based models of decision-making draw substantially on algorithmic models of artificial intelligence and thus rely on associated metaphors of this field. In contrast, the current study avoids metaphors and aims at a first-hand identification of the behavioral elements of a process of choice.

**Method** **:** We designed a game in Mouselab resembling the real-world procedure of choosing a wife. 17 male subjects were exposed to cost-benefit decision criteria that closely mimic their societal respective conditions.

**Results: **The quality of choice index was measured with respect to its sensitivity to the final outcomes as well as process tracing of decisions. The correlation between this index and individual components of process tracing are discussed in detail. The choice quality index can be configured as a function of expected value and utility. In our sample the quality of choice with an average of 75.98% (SD: ±12.67) suggests that subjects obtained close to 76% of their expected gains.

**Conclusion: **The quality of choice index, therefore, may be used for comparison of different conditions where the variables of decision-making are altered. The analysis of results also reveals that the cost of incorrect choice is significantly correlated with expected value (0.596, sig = 0.012) but not with utility. This means that when sub-jects face higher costs prior to making a decision, there exists a corresponding higher expectation of gains, i.e., higher expected value.

Uderstanding the cognitive processes of decision- making helps determine the applied strategy, predict the subsequent decision-making behavior, and its consequences (1, 2). The capability to strike a balance between immediate and long-term consequences of choices is defined as decision-making (3, 4). Collecting information from the respective environment by adopting search strategies is one of the processes of decision making. Such balanced strategies while guarantee the achievement of the goal, would prevent undesired extra entropy (5-7). 

Evidently, more generalized and simpler strategies correspond to lower consumption of cognitive resources (8).

To identify the cognitive processes underlying decision making, one may focus on methods used for collecting information. Therefore, studying information acquisition and consumption can provide material for deduction of the nature of information processing and cognition. 

Tracking down the process of decision-making, the data related to search for information can be used to postulate how the person thinks (9, 10). In other words, the choice of objects goods or gambles reveals hidden preferences.

Theoretical framework 

Behavioral decision research deals with the formulation of theories surrounding cognitive processes, using processing models that describe thinking processes during decision-making or judgment (11, 12). A result-based (structural) model measures the relationship between "attributing'' values and "alternative" options of decision-making (13, 14). In fact, a structural model focuses on the final outcome of a decision and tries to link the final results of various aspects of decision making. So far, the structural model has only been used to measure the relationship between the value of attributes and the final response without referencing to the process of decision making. However, process tracing is an alternative approach with more emphasis on the process of decision-making that focuses on the quantity, type, time, and sequence of information acquisition as well as the evaluation processes (15, 16). 

To date, most studies use these aspects of decision-making in terms of tools and design; some tools measure the final result of decision-making and some measure processes tracing. It is evident that different tools should be developed to measure both decision-making and processes tracing in one setting. 

Several criteria have been developed to examine information acquisition behavior in decision making (12).

**Time of decision making** **:**

The first parameter used to investigate and compare individuals behavior is the time taken to make a decision on each screen or round of a game. 

**Task-based complexity** **:**

The term “task-based complexity” refers to the measurement of common properties of actions of interest in the process of decision making:

1. Number of alternatives and attributes (amount of information(
2. Information display style (sequentially or simultaneously(
3. Response method (selecting or scoring the alternatives(

Several metrics have been developed to examine the pattern of information acquisition in decision-making (12, 17).

Another component of task-based complexity is "choice quality", which is determined by measuring the level of similarity between the choices of a decision-maker and the best matching decisions given same the alternatives (18-21). The basic problem is determining the subjective value of the equation because in addition to the usual subjective biases, the equation suffers from social desirability bias. 

The goal of this study was to develop a tool that could measure both the structural and process tracing techniques, with which comprehensive studies in the field of economics and marketing neuropsychology have been made possible. The other goal of this study was to test as closely as possible the problem-solving situations. Moreover, in this study, the precise time of the actions was measured to extract the possible time of functions. The findings obtained using this tool was applied to evaluate the indices that determine decision-making behavior in uncertain situations or when there is insufficient information.

## Materials and Methods

***Tools***

In this study, the Mouselab software was applied to monitor decision-making attributes and data generation as the main analytical tool. This software was developed by Willemsen and Johnson in 2006 and is a process tracing software that can monitor information acquisition in the decision-making process (10).

The Mouselab, as a computerized process tracing tool, uses actions of the mouse cursor (10, 22). Besides computer-based actions, input in Mouselab is dependent on visual cognition. The Mouselab software is available online at www.mouselabweg.org and its application is subjected to terms of General Public License (GNU). Mouselab avails the technology incorporated in browsers, the HTML dynamic page, and the JavaScript. The function of this software is to register activities and movements of the computer mouse in milliseconds.

The main dialogue of the software is a matrix of alternatives and attributes, where a set of information is assigned to different cells of the matrix. The information is hidden beneath the cells, and each chunk of information is only revealed when the mouse moves over a matrix cell. The software records a set of information about stages of information acquisition, such as the quantity, duration, and sequence of opening of the cells (23). In addition to the situations where the Mouselab software is applied to measure endeavors in decision-making(19, 24-26), this tool may also be used to measure compliance to time-based and cost-based coercive conditions (14, 27).

Moreover, this software is also applied to test the patterns of cumulative data acquisition in multivariate and multi attribute situations (28). In this study, the setting of Mouselab software, similar to its parent versions, focused on collection of the data specific to decision-making behaviors. The default properties of the software are not presented. The new capabilities that the applied setting added to the original capacities of the software has also been described. In this study, a game compatible with the Persian language and easy to understand for the typical participants of this study, was designed. Based on the narrative of the game, 3 young men needed to select a girl, out of 4, to marry (alternatives). Selection had to be in accordance with preference criteria of the young men and based on 4 characteristics of the girls (attributes). The attributes were available in a binary coding as “Yes” or “No.” The game interface was, therefore, designed as a multi-attribute and multivariate matrix of 4 ✕ 4, with the binary data being hidden under the cells and only available at a cost. All the data and sorting were randomized. The game was not a gambling one and each round had an actual correct answer. The participants were trained before playing the game. 

In the setting applied in this study, the cost of each information unit (opening a box) was IRR 450 and each time step was IRR 780. Therefore, the choice was determined by calculating the cost of information acquired (number of opened boxes × IRR 450 = cost of information acquired), as well as the time pressure (number of time steps × IRR 780 = time pressure) to enter the last step of the decision-making processes. If the choice was correct, the participant was provided with a feedback credit bar [IRR 15000 − (cost of information + time pressure)].However, making a wrong choice resulted in losing any reward from that round of the game. In any event, the participant moved to the next round with the remaining credit, which was calculated as the total credit at the beginning of the index round − the amount lost on the intended round ( The primary credit was IRR 900 000 for 60 rounds, which is IRR 15 000 for each round).

Additional feedback properties were provided in the default interface. The default interface already had a bar displaying the passage of time during the index game. The additional feedback included: (a) a feedback bar displaying real-time remaining credit of the specific round of the game based on the data purchasing and lapse of time; (b) a feedback bar showing real-time remainder of the total credit; and (c) immediate feedback on the success or failure according to the final choice at each round. Furthermore, given the 16 cells of the matrix, and the limited capacity of human working memory (29-33), it was decided that, in contrast to the default feature of the Mouselab interface, upon opening of each cell, the purchased data remain available to the player by keeping the cell window open. By this modification, it was assumed that the process of decision-making would be free of the confounding factor of memory capacity. To provide further details, screenshots of the interface of the game, samples of JavaScript, and PHP TEXT of the game, are annexed to this manuscript.

***Participants***

The participants in this study included a group of 17 individuals who were volunteer college students with no history of substance use or psychiatric diagnosis. The mean age of the participants was 33.8 ± 8.83 years and all were male, and Iranian. Also, they had at least high school diploma. The number of participants with higher education or bachelor's degree was 11.

The research proposal was reviewed and accepted by Tehran University of Medical Sciences (TUMS) (IR.TUMS.VCR.REC.1397.465). Participation in this study was on a voluntary basis and required a written consent. As the study method included games with monetary credit, the participants received the exact amount they had gained throughout the game after finishing the task. All the 17 cases fully completed the stages of the study.

  Iranian Rial

## Results

**Figure 1:**
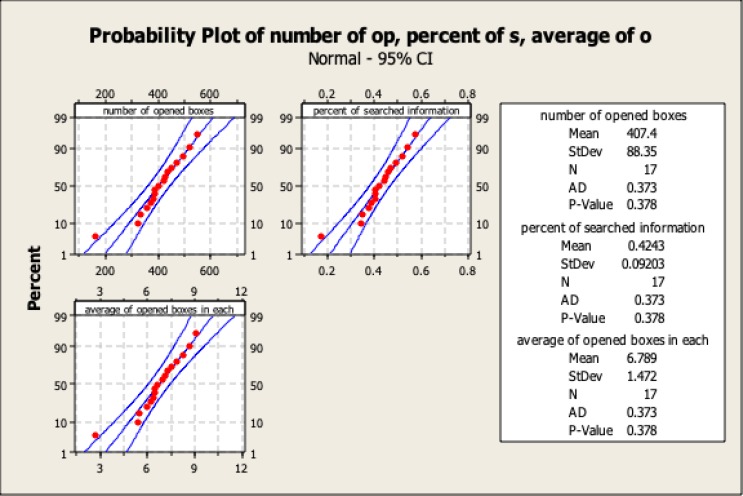
Probability Analysis of the Data Related to Opened Boxes: Number, Percent and Average of Opened Boxes

**Figure 2:**
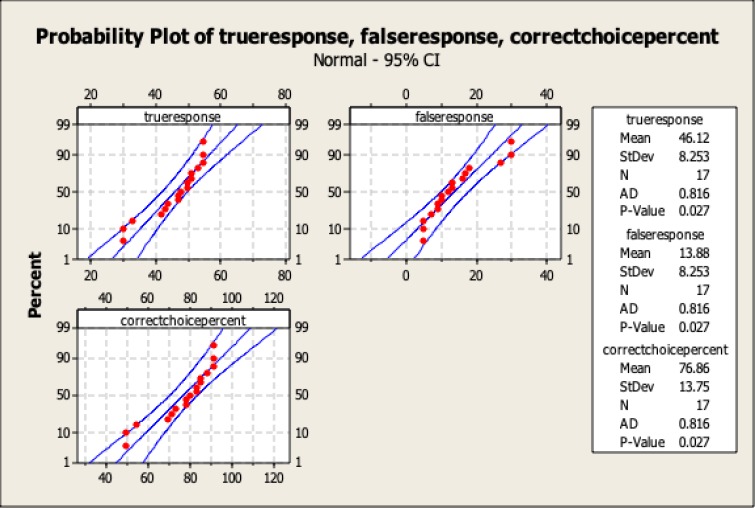
Probability Analysis of Variables Related to Accuracy of Choices

The statistics related to quantity of searched data throughout the game are presented in Table 1 and Figure 1. The figures illustrate the data acquisition per round and per game (60 rounds). The average data used for decision-making were not complete, and on average the participants only acquired 42.43% of the available data. Although the participants did not make their decisions based on complete information, 76.8% of their choices were correct (Table 1 and Figure 2), compared to a random choice, which has a correctness probability of 25% (given that each round consisted of 4 options). 

**Figure3:**
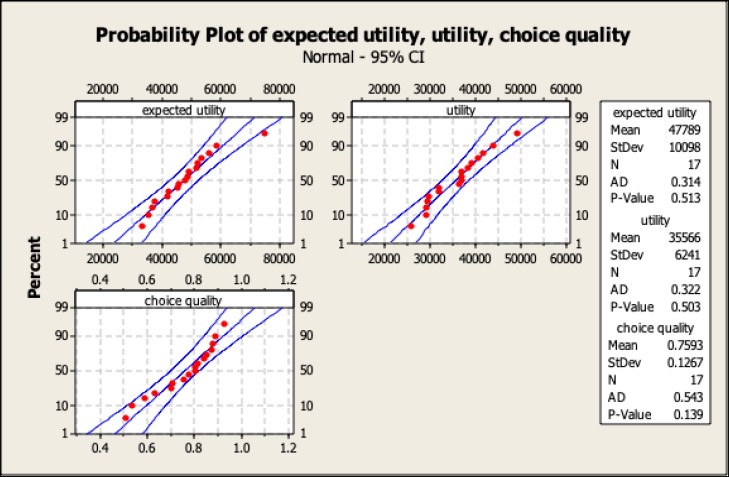
Probability Analysis of Variables Related to Costs of Searches

The cost-based information of the game is presented in Table 2 and Figure 3. The first row represents the mean of the total expected value of all 60 rounds, which is calculated as the total credit per round − cost per round (cost of data purchased + time-lapse). The second row shows the net profit of the game (60 rounds), calculated as expected value − cost of incorrect choices. The last row indicates the “choice quality”, which is the index calculated as (utility / expected value) × 100, according to its theoretical definition.

The participants of this study scored a mean value of choice quality of 75.93% (SD: ±12.67), indicating that the average reward for or satisfaction of each participant was around 76% of the total credit subtracted by the costs of process tracing elements. Table 3 and Figure 3 shows the result of correlation test between the intended variables, which is based on the regressive correlations between the interactive variables. 

The results indicated no meaningful correlation between utility and incorrect choice cost. However, incorrect choice cost presented a relatively high correlation with the expected utility. A significant negative correlation was detected between the expected utility and choice quality, indicating that the faster the player got to the last stage (selecting an alternative), the less the quality of choices.

**Table 1:** Descriptive Analysis of the Data Related to Searched Information and Accuracy of Choices

	Mean(sd)	Median (Interquartile Range)
**Number of opened boxes**	407.35(88.34)	403(95.50)
**%of opened boxes**	0.42(0.04)	0.41(0.10)
**average of opened boxes**	6.78(1.47)	6.71(1.59)
**Correct choice**	46.11(8.25)	48(9.50)
**Wrong choice**	11.88(8.25)	12(9.50)
**Correct choice %**	76.86 (13.75)	80(15.83)

**Table 2:** Descriptive Analysis of Cost-Based Data of the Game

	**Mean(sd)**	**Median(Interquartile Range)**
**Expected utility**	47789(10098)	47829(13134)
**Utility**	35566.23(6241.26)	36744(10270)
**Choice quality**	0.7593(0.1267)	0.8048(0.1938)

**Table 3:** Correlation between the Variables Related to Cost and Accuracy of Searches and Choices

		**Incorrect ** **choice cost**	**Expected ** **utility**	**Utility**	**Opened ** **boxes (%)**
	N	17	17	17	17
**Incorrect choice cost**	Pearson Correlation	1			
	Sig. (2-tailed)				
**Expected value**	Pearson Correlation	0.596*	1		
	Sig. (2-tailed)	0.012			
**Utility**	Pearson Correlation	-0.348	0.521*	1	
	Sig. (2-tailed)	0.172	0.032		
**Opened boxes (%)**	Pearson Correlation	-0.737**	-0.802**	-0.120	1
	Sig. (2-tailed)	0.001	0.000	0.645	
**Correct choices (%)**	Pearson Correlation		-0.601*	0.328	0.724**
	Sig. (2-tailed)		0.011	0.199	0.001
**Choice quality**	Pearson Correlation		-0.596*	0.349	0.717**
	Sig. (2-tailed)		0.012	0.170	0.001
					

* ***p* value < 0.05**

** ***p* value < 0.01**

## Discussion

This study aimed to explore the nature and mechanism(s) of decision-making based on an ecological point of view that closely approximates the real-world decision-making situations by addressing reason and rationality of behavior. A language fit arrangement of the Mouselab software was used in this study. In addition to the features of a normal process tracing tool, the mentioned arrangement provided features for more precise measurement of the choice quality as a function of expected utility and utility. Considering the data obtained using this adapted setting of Mouslab software, it seems that expected utility is a product of interrelation between properties of the sum of the total scores of the rounds up to a specific point of the game and the immediate feedback of the remained credit at that given point (see the negative correlation between expected utility and correct choice as well as the opened boxes).

In this study, decision-making was addressed in a results-based structural model with focus on the final outcome of decision by measuring the defined goals and focusing on the sequence of information acquisition and evaluation processes. This was achieved through adaptation of the settings of the software by adding new capacities, including extra feedback as well as measuring process tracing based on real-time quantity of the reward. The results of this study revealed that the participants demonstrated similarities in their choice quality under time pressure and stress conditions, paired with different levels of time and effort.The findings of this study are in line with the argument raised by Gigerenzer et al (2011) that complete or close to complete information not only results in ambiguity in a perfect decision-making, but also a perfect decision is more dependent on cue-based information (34). Patterns of information acquisition have a direct impact on cognition and memory. Therefore, contextual changes in presenting information can alter the frequency of preference reversal (35), choice strategy, and decision performance (36).

In this study, the proportion of the utility to the expected utility is defined as “choice quality.” Choice quality of a decision is a function of consumption of resources and the accuracy of the outcome represented by quantity of reward. This index does not simply represent the immediate outcome of a decision, which is the total reward gained by the player; it rather shows the relationship between the cost of resource consumption (information and time) and confidence of the player in her/his choice. Apparently, two determinants of choice quality are expected utility and utility that, by definition, are the 2 components of the outcome. In fact, expected utility appears to be a dynamic quality that requires repeated rounds of the game. 

Therefore, choice quality, as defined in this study, can be used when the costs of process tracing and the rewards of decision-making are of non-homogeneous quality or of different scales. Furthermore, choice quality would be a valuable measurement for comparing decision-making in different types of games or games with different rounds.

The focus of a result-based evaluation of a decision-making study would be on the penalty of wrong choice, which is calculated as expected value − utility. However, utility/ expected utility is a result-based approach, and process tracing approach reflects a more precise index of accuracy of choice. The accuracy of decision-making or “quality of choice”, therefore, can cover both the result-based and process tracing interests. In other words, the ratio of utility/ expected utility demonstrates the accuracy of the whole process of decision-making because it reflects the impact of all components of the game and covers them as a whole (time consumption, quantity of data purchased, correctness of choices, expected utility, and utility of the subject). The choice quality or accuracy of decision index was calculated for all the participants and the results are presented in Figure 3. In fact, the choice quality index reflected the satisfaction of the subject of his decision. 

## Limitation

The most notable limitation of the present study was the relatively small sample size of participants. Also the validity of driven constructs must be assessed by comparing of them with their counterparts driven by valid tests. This will be done using the IOWA Gambling and Tower of London tests. 

## Conclusion

As a psychometric tool, the Mouselab software has been used to statistically describe decision-making phenomena, both structure-wise and process-wise. By applying data mining techniques, the software sheds light on strategies and paths that individuals use and generate during the decision-making processes. Behavioral economists and psychologists have mainly used inferences from decision-making process data to identify the strategies and paths of the process or structure of a decision. The focus of these studies has been on the scale of efforts that lead to a decision. Therefore, the choice quality of a decision has only been indirectly and imprecisely defined and calculated (16, 20, 37-44). However, the main contribution of this study is providing a direct measure of the choice quality of a decision.

Despite the possible limitations of this exploratory study, the findings of the present study have implications for future orientation in decision-making and judgment studies, in particular in behavioral economics, decision psychology, and neureconomics. For instance, it may provide grounds for comparing decision-making in different groups of samples with a specific property, such as a disease (eg, addiction), or with a control group, or making comparisons with the decision made on a computer or in machine learning.
